# Variation in hunting behaviour in neighbouring chimpanzee communities in the Budongo forest, Uganda

**DOI:** 10.1371/journal.pone.0178065

**Published:** 2017-06-21

**Authors:** Catherine Hobaiter, Liran Samuni, Caroline Mullins, Walter John Akankwasa, Klaus Zuberbühler

**Affiliations:** 1 Centre for Social Learning and Cognitive Evolution and Scottish Primate Research Group, School of Psychology and Neuroscience, University of St Andrews, St Andrews, Scotland; 2 Budongo Conservation Field Station, Masindi, Uganda; 3 Department of Primatology, Max Planck Institute for Evolutionary Anthropology, Leipzig, Germany; 4 School of Veterinary Medicine and Animal Resources, Makerere University, Kampala, Uganda; 5 Department of Comparative Cognition, Institute of Biology, University of Neuchatel, Neuchâtel, Switzerland; University of Portsmouth, UNITED KINGDOM

## Abstract

Hunting and sharing of meat is seen across all chimpanzee sites, with variation in prey preferences, hunting techniques, frequencies, and success rates. Here, we compared hunting and meat-eating behaviour in two adjacent chimpanzee communities (*Pan troglodytes schweinfurthii*) of Budongo Forest, Uganda: the Waibira and Sonso communities. We observed consistent between-group differences in prey-species preferences and in post-hunting behaviour. Sonso chimpanzees show a strong prey preference for Guereza colobus monkeys (*Colobus guereza occidentalis*; 74.9% hunts), and hunt regularly (1–2 times a month) but with large year-to-year and month-to-month variation. Waibira chimpanzee prey preferences are distributed across primate and duiker species, and resemble those described in an early study of Sonso hunting. Waibira chimpanzees (which include ex-Sonso immigrants) have been observed to feed on red duiker (*Cephalophus natalensis*; 25%, 9/36 hunts), a species Sonso has never been recorded to feed on (18 years data, 27 years observations), despite no apparent differences in prey distribution; and show less rank-related harassment of meat possessors. We discuss the two most likely and probably interrelated explanations for the observed intergroup variation in chimpanzee hunting behaviour, that is, long-term disruption of complex group-level behaviour due to human presence and possible socially transmitted differences in prey preferences.

## Introduction

Hunting and meat sharing is regularly observed in wild chimpanzees at all long-term study-sites [1–8]. Mammalian prey species recorded have included other primates, ungulates, and rodents [8–12]; however, prey preferences, hunting techniques, frequencies, and success rates, as well as the degree of active and passive meat sharing, can vary considerably between sites [2, 3, 13–16]. As ominvores, chimpanzees have a broad diet and meat makes up a relatively small portion of it [17–19]. Given the range of dietary alternatives and the potential risks of significant injury following a fall from the canopy while chasing primate prey [14], the prevalence of group (as opposed to solo) hunting, and the variation in hunting behaviour across chimpanzee groups requires explanation. Group hunting is relatively common in social carnivores (for example in: lion, *Panthera leo* [20]; spotted hyena, *Crocuta crocuta* [21]; killer whales, *Orcinus orca* [22]), which are dependent on meat for the majority of their calorific needs. Among Taï forest chimpanzees there is evidence that pay-off rates increase per capita for group hunting [14, 18]. However, total calorific returns appear to vary between sites and, as alternative fallback foods are typically available, others have proposed that access to micronutrients drives hunting in chimpanzees–the ‘meat-scrap hypothesis’ [23, 24].

After hunting chimpanzees actively share both small and large pieces of meat with unrelated adults, both within [25] and outside of a sexual context ([4]; but see also [15]). Tolerated co-feeding or recruitment to a feeding site has been reported across a wide range of species (e.g. killer whales [22]; chickens [26]; ravens [27]; puma [28]). Active food sharing, however, is more typically restricted to mating pairs or family groups or to sexual contexts (e.g. the nuptial gifts of male insects [29, 30]; or bird ‘helpers at the nest’ [31, 32]). Both between close-kin and in sexual contexts the fitness benefits of active sharing are evident for both the donor and recipient; however, interesting exceptions outside of these contexts have been reported in some species (bonobos [33, 34]; vampire bats [35, 36]; jackdaws: [37]), including chimpanzees [4, 25). The frequency of active (and passive) food sharing varies between chimpanzee groups and may be influenced by social factors such as harassment [38, 39] or presence of oestrus females or social allies [4, 40–45].

Variation in behaviour between populations, as seen in the hunting and meat-sharing differences among chimpanzee communities, has been of particular interest to the ongoing debate on the evolution of human culture [46]. One way to identify potential cultural traits, or behavioural traditions, is to compare populations of the same species and focus on behaviour that is performed by a number of individuals over prolonged periods of time within one group or population but is absent in another [47], provided there are no obvious genetic or ecological differences between the two study groups (the ‘exclusion’ method, [17]). Although this exclusion method has a number of inherent problems (e.g. [48]), it continues to provide the thrust for the argument that chimpanzees have a capacity for culture. To date, the evidence supporting cultural variation between chimpanzee groups is biased towards differences in tool use and other non-social foraging related behaviour [46]; however, hunting and subsequent meat eating provides a social foraging context in which to investigate this question.

In this study, we were interested in the potential differences in a group-level behaviour–hunting–and to what extent these could be the result of ecological and/or cultural factors. There are currently two impediments to considering differences in chimpanzee hunting behaviour as cultural. Firstly, the currently available observational records stem from two different sub-species (*Pan t*. *schweinfurthii/verus*) from East and West Africa. And secondly, even within sub-species, there remains considerable ecological variation between sites [40, 49], making it difficult to rule out non-cultural explanations for differences [48]. Even within sites, assessing ecological factors in a complete manner is challenging–fine-grained analyses of ‘microecological variables’ may reveal alternative ecological explanations (e.g. adaptation to prey behaviour, [50]), and both cultural and ecological factors may interact [51]. In assessing ecological factors that impact chimpanzee hunting, studies to date have typically focused on group composition or social structure [23, 43, 52], and forest canopy structure [11, 18]; however, another possible source of variation–human presence–remains unexamined, despite its potential impact on behaviour [53].

Here, we present the first observations of hunting and meat-eating behaviour in a newly habituated group, the Waibira community of Budongo Forest, Uganda, and compare them with long-term data of the neighbouring Sonso community. Both groups share the same forest environment and there is regular genetic flow between the groups. We are able to compare and contrast the behaviour of the two communities at a similar stage in habituation to human research presence using long-term records for the Sonso community and comparing with published records from Sonso and other communities. Where all of these factors can be excluded, any remaining group-level behavioural differences become a candidate for a socially acquired ‘cultural’ variant. Fast-paced hunts are often high in the canopy and can be difficult to observe, even among well-habituated chimpanzees; however, prey species and subsequent meat-eating behaviour can be recorded accurately and reliably after the kill, and are the focus of this study.

## Method

The Budongo Conservation Field Station (BCFS, formerly the Budongo Forest Project) was established in 1990 in the Budongo Forest Reserve, situated in the western Rift Valley of Uganda. The 793-km^2^ reserve includes 482 km^2^ of continuous, medium-altitude, semi-deciduous forest cover [54] with an estimated population of around 600 chimpanzees [55]. Regular daily observation of the Sonso chimpanzee community started in 1991, with long-term data available from 1994, while habituation of the Waibira community started in March 2011. In both groups, periods of systematic focal behaviour sampling (including party composition, ranging, and activity behaviour) have been complemented with all occurrence data recording of the frequency and duration of unusual events, including hunts. In the Waibira group, data were collected using a handheld HP iPaq PDA and a Panasonic HDC-SD60 camcorder. At the time of writing (April 2017), 95 Waibira group members had been individually identified (≥ 12 years: 26 males, 31 females). The community probably has more adult females as males habituate more quickly to human observers than females [6, 56], suggesting an estimated group size of around 100–120 individuals. At the time of writing, the neighbouring Sonso community consisted of 69 individually identified group members (≥12 years: 11 males, 31 females). Rank relations between group members were determined through ad libitum observations of pant-grunt calls, a vocal signal emitted by subordinates upon encountering a higher-ranking group member [2, 57].

### Data collection

#### BCFS long-term data

A permanent staff (currently n = 8 field assistants; n = 4 in each community) at the Budongo Conservation Field Station collect focal behavioural data and party composition data on a daily basis. Schedules are rotated to cover all days except public holidays. Typically 3 field assistants are present in each community each day. Working hours are 06:00 to 18:00 in the Waibira community and 07:00 to 16:30 in the Sonso community; however, travel times to reach the Waibira range are around 45-60min so chimpanzee contact time is similar in both communities, and data are typically recorded between 07:00 and 16.30 local time. Where individual research projects require it nest-to-nest follows in which data area recorded between 6:00 and 19:00 are also conducted in both communities. Long-term data currently requires continuous recording of all behaviour from a single focal individual in combination with scans of party composition and behaviour every 15min throughout the day. For both groups, events books are kept by researchers and field assistants to document all unusual or otherwise remarkable behaviour, including hunting and food sharing. Hunting behaviour is recorded on an ad libitum basis and includes all observations of hunting and meat eating (whether or not a focal individual was involved). For the Sonso community, detailed event book entries were available from Jan 1999—February 2017. For the Waibira community, entries were from April 2011- February 2017. Additional information was obtained from interrogating two long-term field assistants, who have worked with the Sonso community (Geresomu Muhumuza: 26-years, Monday Gideon: 20-years) and from a specific study of hunting behaviour conducted between 2010 and 2012 (see below). Finally, data from a study of Sonso prey preferences between 1994–2002 [5] are included for comparison.

Unidirectional pant-grunt data were collected both as part of the systematic long-term data collection and independently within the specific study of hunting behaviour. These are used to construct the male hierarchy. It is assumed that all independent mature males are ranked above any female and immature individuals, and that the hierarchy is linear amongst mature males (previous research has found that the Sonso community has a steep ‘despotic’ (rather than egalitarian) hierarchy structure, [58]). We are cautious in our interpretation of rank relationships between mature Waibira males as the very large number of independent males means that they are regularly spread across parties and may not be recorded within the same party for weeks or even months, during which time there may have been changes in their relative ranks. However, as in Sonso, it is assumed that all independent mature males are ranked above any female and immature individuals.

#### Specific study of hunting behaviour in 2010–2012

Focal individuals within this study were 13 male chimpanzees aged 10years and older (total focal hours = 361.2; mean hours per focal = 27.8 ±9.8). While following a focal individual all hunting and meat-eating behaviour from any member of the party was recorded on an all-occurrence basis, taking point samples from any individual when a new behaviour was observed [59]. Focal individuals were selected in a pseudo-randomised order and full day follows were conducted (7:00–16.30) to avoid biases in data collection based on time of day, general activity, or location. Pseudo-random focal selection was used, where individuals in a party were initially searched for in the morning according to a random schedule and later chosen according to which (within the party) was the most under-sampled within the existing data set, due to the fission-fusion group dynamic and long-ranging behaviour of individuals [60].

#### Hunting data collection

Individuals were classified as hunters if they carried out any behaviour where they were following the prey, either on the ground or in the trees. This included those individuals observing from the ground, as per Watts and Mitani [11], which have also been described as bystanders by Boesch [10]. Group hunts were classified as any hunting behaviour involving two or more individuals. Behaviour recorded from hunters included: party composition, active role as hunter (chaser, blocker, observer as per [10]), location on the Budongo grid-system (100mx100m transects) and social interactions between chimpanzees (including feeding and meat sharing behaviour if prey were killed, and any additional notes).

Data were tested for appropriateness for parametric analysis (normality and homogeneity of variance). Where data were not suitable, non-parametric alternatives were used. In the case of non-parametric tests, to avoid pseudo-replication, we analysed each behavioural event (e.g. aggressive attack of another individual, sniffing meat without feeding, dropping the carcass un-eaten) only once per hunt, irrespective of whether or not it was reported for multiple individuals. For example, if a carcass was picked up and dropped multiple times by different individuals in a hunt we counted this as a single occurrence of ‘carcass dropping’.

Sharing behaviour was assigned to one of two categories during analysis of video and written records. ‘Active sharing’ was defined as the individual in possession of the meat handing over to (using hands, feet, or mouth) or dropping a piece of meat in front of another individual. ‘Passive sharing’ was defined as tolerating other(s)’ feeding from the meat that remained in the individual’s possession. Harassment includes persistent peering and begging [38, 39, 61]; however, within this we specify *aggressive harassment* as harassment that included dominance or threat displays (indicated by behaviour such as piloerection, swaggering and galloping, dragging objects, and vocalizations such as barks or screams), chasing, or physical attack.

#### Prey species survey

Estimating mammal densities along line transects using both direct observation and observation of indirect signs (e.g. dung) are established methods to provide reliable data when used with the appropriate estimations of error [62, 63]. We surveyed 20km of line transects covering the home ranges of both communities. Ten 2km long parallel 2m wide transects were marked along established trails running east-west (10km in Sonso, and 10km in Waibira). Stratified random transects were distributed at approximately 400m north-south intervals and included all three major secondary forest-types present in the Budongo Forest reserve (swamp, mixed, climax). All trails were walked once in February 2016 and once in February 2017 at speeds of approximately 1km/h, starting between 7-9am.

Non-primate large mammal abundances were estimated from the standing crop of dung. Within dense secondary rainforest observation conditions can be poor, particularly for unhabituated mammals, requiring that dung counts be used as an estimate of abundance rather than specific population density [64]. Species differences between red-duiker and bushbuck could not be reliably identified from indirect observations of dung so these data were combined. Primate densities (red-tailed monkey, blue monkey, black and white colobus) were estimated from direct observation of individual groups [62].

For all species we recorded the species name and the GPS location of dung/group, then calculated the Mean Encounter Rate (MER) and standard error. For direct observation of primate groups we recorded the perpendicular distance and height from the observers (both estimated from the centre of the party). Densities were calculated using DISTANCE software v7 [65]. Mean adult body mass of prey species in kilograms was taken from Kingdon [66] with male and female mass indicated separately where there is sexual dimorphism.

### Ethical note

This study was purely observational and did not involve any interventions, apart from daily visits to the two study communities. Researchers and field assistants follow strict hygiene, quarantine, and observation distance rules to prevent disease transmission, as detailed in the project guidelines (www.budongo.org). Permission to study the chimpanzees has been given by the University of St. Andrews Animal Welfare and Ethics Committee, as well as the Uganda National Council for Science and Technology, following the advice of the Uganda Wildlife Authority, and National Forest Authority.

## Results

### (a) Hunting behaviour: Prey species hunted and success rates

#### Sonso

The Sonso chimpanzees fed on the meat of six species, including four primates, following 203 hunts, 182 of which were successful over a 17-year observation period (89.7% success; Table 1). These numbers are an underestimate, partly because it is impossible to document all hunting events throughout the year. This is particularly true for unsuccessful hunts, which are more difficult to document. When we examined data from a 22-month period (January 2010 to October 2011) in which CM collected all evidence of hunting activity during bouts of focal animal sampling, we found significantly lower success rates (focal sampling hunt success: 8/19 hunts, 42.1% success; long-term data 182/203, 90%, p<0.0001, 2-tailed Fisher’s exact test).

**Table 1:** Specialization of hunting in the Budongo chimpanzees, 1999–2017.

	Mean adult body mass (kg)	Sonso (1999–2017)		Sonso 1994–2002*	Waibira (2011–2017)	
Species		attempted n (%)	successful n (%)	successful n (%)	attempted n (%)	successful n (%)
Guereza colobus monkey (*Colobus guereza occidentalis*)	10–23	151 (74.4)	137 (75.3)	7 (41.2)	13 (36.1)	7 (23.3)
Blue monkey (*Cercopithecus mitis stuhlmanni*)	3.5–5.5(f)5.5-12(m)	23 (11.3)	20 (10.9)		3 (8.3)	3 (10.0)
Red-tailed monkey (*Cercopithecus ascanius schmidti*)	1.8-4(f) 3-6(m)	7 (3.4)	7 (3.9)		2 (5.6)	2 (6.7)
Unconfirmed *Cercopithecus sp*.	-	2 (0.9)	2 (1.1)	5 (29.4)	0	0
Olive baboon (*Papio anubis*)	11-30(f) 22-50(m)	4 (2.0)	3 (1.6)	0	0	0
Blue duiker (*Cephalophus monticola*)	3.5–9	14 (6.9)	12 (6.6)	4 (23.5)	9 (25.0)	9 (30.0)
Red duiker (*Cephalophus natalensis*)	12–14	1 (0.5)	0 (0.0)	0 (0.0)	9 (25.0)	9 (30.0)
Elephant shrew (*Rhynchocyon cernei*)	0.04–0.05	1 (0.5)	1 (0.6)	1 (5.9)	0	0
**Total**		**203**	**182**	**17**	**36**	**30**

Results are displayed as number of each species hunted, followed by the percentage that this represents of all animals hunted in parentheses. Adult body mass of prey species is marked in kilograms [66] with male and female mass separate where there is sexual dimorphism. Events per species are given for both observed attempts (includes all hunting activity irrespective of success) and successful hunts (successful hunts include events where the hunt was not observed but the subsequent meat sharing was). Note: as hunting behaviour was not studied directly until 2008 unsuccessful hunts frequently went unobserved, and even when observed were unreported. Data from unsuccessful hunts are reported in order to provide additional information on the frequency with which different species were hunted, rather than as an accurate indication of hunting success. *Data taken from Newton-Fisher et al. [5]; only successful hunts were reported.

Guereza colobus monkeys (*Colobus guereza occidentalis*) constituted 74% of all prey items, significantly more than all other species combined (attempted hunts: p<0.0001; successful hunts: p<0.0001; binomial tests, two-tailed), indicating a clear bias towards this species (see Table 1). On a single occasion Sonso chimpanzees were observed to chase a red duiker (*Cephalophus natalensis*), apparently with the intent of capturing it, but the attempt was unsuccessful. Juvenile and infant chimpanzees were observed to interact playfully (chasing without aggression or attempts to kill or injure) with both red and blue duikers (*C*. *monticola*), as well as to groom and play with three monkey species: blue monkeys (*Cercopithecus mitis*), red tail monkeys (*Cercopithecus ascanius*), and olive baboons (*Papio anubis*).

87.6% of Guereza colobus monkey hunts involved multiple chimpanzees (N = 89 records with sufficiently detailed information on identity of hunters). Solo colobus hunts, while rare (n = 11), were not less successful than group colobus hunts (n = 78) (success rates: solo hunts: 9/11; group hunts: 60/78; p = 1.00, 2-tailed Fisher’s exact test). In non-colobus prey species (three primates and blue duiker; N = 24 records with detailed information on identity of hunters), solo hunts were significantly more common than for colobus prey (solo rates: non-colobus: 22/24, colobus: 11/95; p<0.0001; 2-tailed Fisher’s exact test). If analysing non-primate species only, 100% of hunts were solo (n = 10).

#### Waibira

Over a five-year observation period, members of the Waibira community were observed to hunt successfully or were found eating meat on N = 30 occasions: seven cases of Guereza colobus (23%), three blue monkey (10%), two red tailed monkey (7%), and eighteen duikers (9 red duikers (30%); 9 blue duikers (30%)). We observed four short chases after Guereza colobus monkeys by single males that appeared to be attempted solo hunts, and two group chases but these were unsuccessful. Unlike Sonso, successful hunts in Waibira were biased towards non-colobus species, (p = 0.005, two-tailed binomial test) whereas attempted hunts showed no bias in either direction (p = 0.13, two-tailed binomial test). Comparing the two study sites, the relative frequency of hunting duiker (both species combined) as opposed to colobus was significantly different between the Sonso and Waibira communities, considering both successful (p<0.001, 2-tailed Fisher’s exact test) and attempted hunts (p<0.001, two-tailed Fisher’s exact test). However, when compared with an early study of Budongo chimpanzees (1994–2002, habituation started in 1990), the distribution of colobus to non-colobus prey was similar to that in Waibira (2011–2017): successful hunts n = 17 in early-Sonso data, n = 30 in Waibira, colobus prey n = 7 in both cases. Among non-colobus prey both groups hunted both other primates and duiker (early Sonso n = 59%; Waibira n = 77%), although only Waibira were observed to feed on red duiker (n = 9).

### (b) Rates of hunting in Sonso

Given the bias towards reporting successful hunts described in section (a) and other possible confounds in a long-term data set such as observer bias or experience, and the effects of chimpanzee habituation, we are cautious in exploring ‘rates’ of hunting in the long-term data set. For example: if we apply the success rate from the 22-month specific study of hunting (42%) to the long-term data set (n = 182 successful hunts) this would predict n = 433 hunts over the 17-years, more than twice the n = 203 actually reported. The hunting rate before the correction would be 11.9 times a year (roughly once a month); after it would be more than double that at 25.5 times a year.

Moreover, hunting rates may have changed substantially over the years. Newton-Fisher et al. [5] report a successful hunting rate of n = 17 in 8-years, or 2.1 hunts per year. If we again estimate a success rate of 42%, we obtain a rate of 5.1 total hunts per year. We can compare this with the section of long-term data that follows immediately on from this period (14-years, 2003–2017, n = 175 successful hunts), a rate of n = 13 successful hunts per year, or (with a 42% success rate), an estimated n = 30 total hunts per year, which corresponds to an increase of 6-times that reported in the earlier period. Nevertheless there may still be large year-to-year variation in the annual hunting rate; only 11 hunts were recorded in 2015, and only 3 hunts in 2016, a sharp decline from the 19 recorded in 2014, or the 17 recorded in 2013.

**Fig 1:**
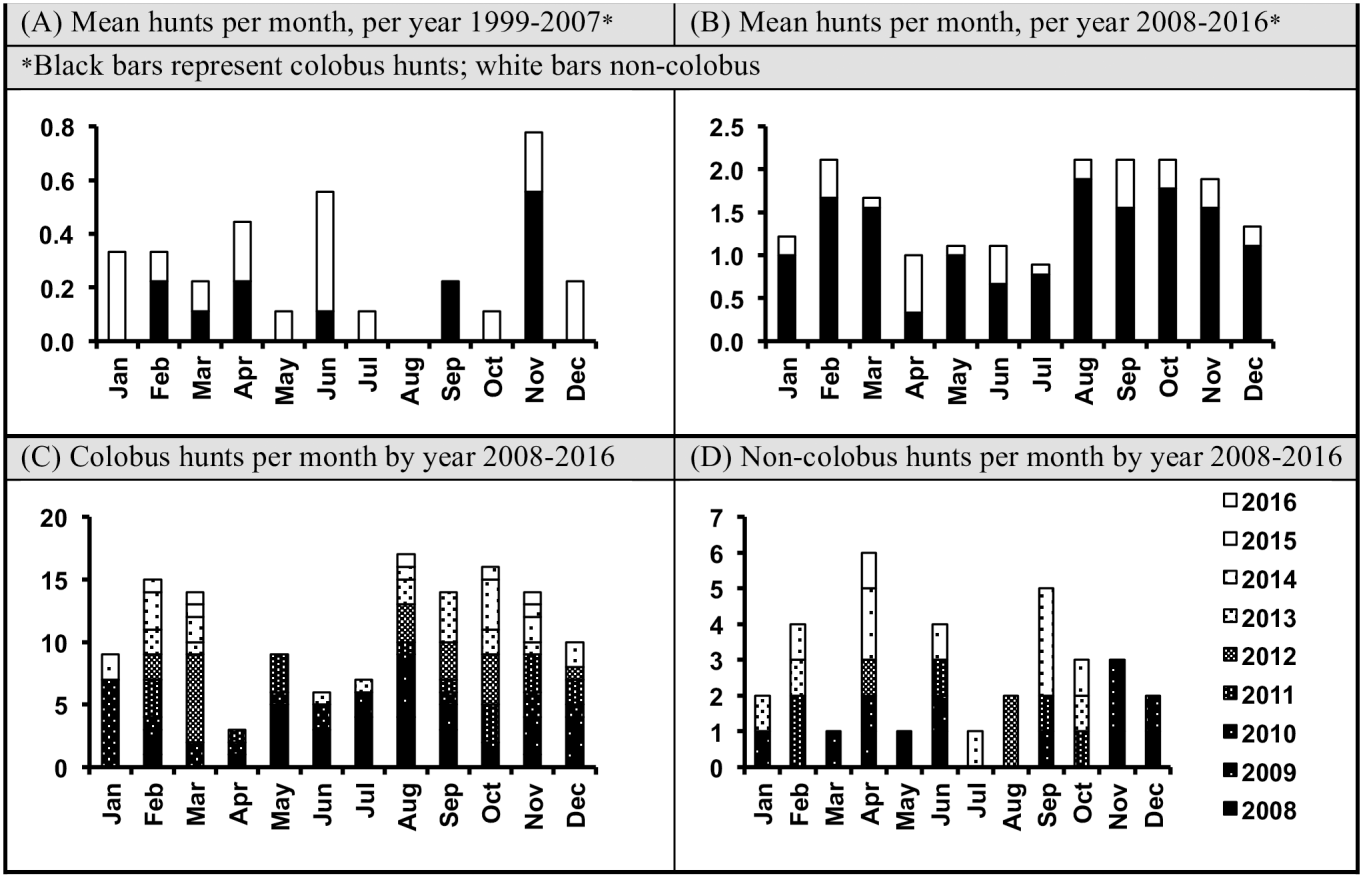
Variation in month-to-month hunting of colobus and non-colobus prey by the Sonso chimpanzees. Monthly rates in panel A and B were calculated by calculating the hunting rate per month for each year, and then averaging across these to control for year-to-year variation within the data sets. Panel A shows the hunting behaviour in the 9-year period from 1999–2007 inclusive; Panel B shows the hunting behaviour in the 9-year period from 2008–2016 inclusive. Panel C and D show the colobus and non-colobus hunting behaviour respectively in the 9-year period from 2008–2016 for each individual year (note that here the axis represents total hunts per month across the 9 years, rather than mean hunts per month, to allow year to year comparison).

Within a given year, we can look at hunting rates from month-to-month. Given our findings above we were extremely cautious about drawing conclusions across years. Instead, we tested for variation on a larger scale by comparing the pattern of hunting month-to-month during the first 9-years of our full data set (1999–2007) with the subsequent 9-years of our full data set (2008–2016). We found that the distribution of hunting frequency month-to-month varied between the earlier and later data on hunting in Sonso for colobus prey (chi square: Χ^2^ = 29.79, df = 11, p = 0.002) but not for non-colobus prey (chi square: Χ^2^ = 11.86, df = 11, p = 0.375) between the earlier and later data on hunting in Sonso. As indicated above, however, when we examined the trends from year to year we see striking differences, so that while the period from April–Jul appears to represent a period of relatively low hunting frequency over the past 9-years (see Fig 1, panel B), we see that in 2008 this pattern was reversed, with the majority of hunting that year recorded between May and August (Fig 1, panel C).

### (c) Abundance and density of potential prey species

The Mean Encounter Rate for mammals, including both primate and non-primate species, monitored appeared higher in Sonso than in Waibira; however, the only statistical difference we found was between blue monkey populations, which appear higher in Sonso (see Table 2). Although Red duiker and Bushbuck were combined here due to difficulties in reliably distinguishing dung, Red duiker were seen on n = 5 occasions in Sonso and n = 4 occasions in Waibira; and Bushbucks were seen on n = 4 occasions in Sonso and n = 1 occasion in Waibira during the survey. Population densities of the three primate prey-species appeared higher in Sonso than in Waibira (see S1 Table); however, as the coefficients of variation (CV) were high (CV range = 17.4 to 39.6%) the sample sizes were likely too small for reliable estimation of density.

**Table 2:** Mean encounter rate of potential prey species.

Species	Sonso MER MER n/km (se)	Waibira MER n/km (se)	Site differences
Blue duiker * (*Cephalophus monticola*)	4.8 (1.2)	4.3 (1.1)	U = 47.0, Z = -0.227, p = 0.82
Red duiker * (*Cephalophus natalensis*) and Bushbuck (*Tragelaphus scriptus Pallas)*	2.4 (0.6)	2.5 (0.6)	U = 45.5, Z = -0.342, p = 0.73
Bushpig (*Potamochoerus larvatus*)	3.4 (0.7)	3.5 (0.7)	U = 48.5, Z = -0.114, p = 0.91
Blue monkey * (*Cercopithecus mitis stuhlmanni*)	2.2 (0.4)	0.8 (0.2)	U = 15.0, Z = -2.685, p = 0.007
Red-tailed monkey * (*Cercopithecus ascanius schmidti*)	1.0 (0.2)	0.9 (1.1)	U = 48.5, Z = -0.118, p = 0.91
Guereza colobus * monkey (*Colobus guereza occidentalis*)	0.6 (0.2)	0.5 (0.1)	U = 45.0, Z = -0.418, p = 0.68

Chimpanzee prey species are marked with an *. In addition, bushbuck and bush pig–two species regularly exposed to human hunting pressure–are included. Abundances are described by the Mean Encounter Rate (MER) of dung per km for non-primates and the MER of groups per km for primates; site differences are tested using the Mann Whitney U test.

### (d) Meat-eating: Monopolization and sharing

#### Sonso

When feeding on colobus meat, more dominant individuals within the party typically took and monopolised possession of the majority of the meat, irrespective of the identity of the hunters. Monopolisation of a carcass by an individual in the presence of a higher-ranking, unrelated individual without aggressive harassment was very rare (N = 3/142 cases) and always involved less desired prey species caught in solo hunts (red-tailed monkey, blue monkey, and blue duiker). In all three cases other individuals begged successfully for access. Sonso individuals who actively participated in the hunt (i.e. were involved in chasing the prey) were not significantly more likely to retain meat (n = 42/118, 36%) than non-hunters (n = 35/120, 29%; total focal observation sample points n = 238; Pearson chi-square test; chi = 1.23, df = 1, p = 0.29). However, individuals who were responsible for the final kill (‘catcher’) were significantly more likely to retain some meat (n = 26/34, 76%), as opposed to losing all possession, when compared to hunters not responsible for the final kill (n = 36/158, 23%; total focal observation sample points n = 192; Pearson chi-square test; chi = 36.9, df = 1, p<0.001), for example by removing the intestines and dropping the remaining carcass.

Feeding on non-colobus prey elicited less apparent excitement or harassment than colobus prey. Instead, we recorded relatively more cases in which non-owners sniffed the carcass without begging or trying to feed (sniffing only: non-colobus: 5/34 instances; colobus: 1/109 instances; p = 0.003; 2-tailed Fisher’s exact test). A similar pattern was observed for individuals dropping all or most of the carcass largely uneaten without harassment (non-colobus: 4/34 instances; colobus: 2/109 instances; p = 0.029; 2-tailed Fisher’s exact test).

#### Waibira

Observations of individuals’ participation in the hunt have only been possible in eight of the 36 cases (attempted and successful); however, during subsequent meat-eating Waibira chimpanzees showed high levels of excitement when feeding on the meat of both monkey and duiker species, with more distant group members rushing to the area after hearing prey alarm calls of both prey types. We recorded persistent begging in all cases, with no observations of sniffing and rejecting meat, or dropping uneaten carcasses for either colobus or non-colobus prey. We also observed no obvious rank effects. All individuals responsible for the final kill were able to obtain meat and maintain its possession. We also recorded instances of both active (within n = 6 hunts) and passive (within n = 14 hunts) meat sharing, following persistent gestural and vocal begging, peering, and touching of the meat.

In contrast to Sonso we observed little aggressive harassment, even when the possessor was in the presence of individuals higher-ranking than themselves (for example: we observed begging gestures produced by the alpha male without aggression towards a sub-adult female). In the thirteen cases in which the possessor was in a party with individuals of higher rank than themselves we observed only two cases of aggressive harassment that led to an individual in possession of a carcass surrendering it: one red and one blue duiker were surrendered to the alpha male (aggressive harassment from higher-ranking individuals Waibira = 2/13 instances, Sonso = 139/142 instances, p = 0.001; 2-tailed Fisher’s exact test).

## Discussion

Although the availability of data from the Waibira chimpanzee community is limited by their recent habituation, we find a current difference in the hunting behaviour of two neighbouring chimpanzee communities in Budongo Forest, Uganda. Sonso chimpanzees show a clear preference for Guereza colobus monkeys, both in terms of their hunting activity and subsequent meat-eating behaviour. In contrast, Waibira chimpanzees show no such preference for colobus, either in hunting or meat-eating, instead catching duiker with equal frequency, including red duiker a species never observed to be hunted by the Sonso community. Furthermore, while high-ranking Sonso chimpanzees typically obtain and maintain possession of meat, irrespective of their own role during the hunt, Waibira chimpanzees, even sub-adults of low relative rank, appear to be able to maintain their possession when in the presence of other higher-ranking individuals while eating both colobus and non-colobus prey.

### Variation in prey-species hunted

At many chimpanzee study sites, hunting efforts appear biased to red colobus monkeys (*Piliocolobus spp*.), including areas where Guereza colobus monkeys are also present [3, 67, 68]. It appears that in the absence of red colobus, which are not present in the Budongo Forest, Sonso chimpanzees have instead specialized in hunting Guereza colobus monkeys. They show a clear bias, in terms of the frequency of both attempted and successful hunts. Feeding on the meat of other species appears less preferred, with individuals even discarding the carcass uneaten.

In contrast, the first observations of the Waibira chimpanzees show no such bias: in addition to Guereza colobus, they hunt duiker with equal frequency and show similar social behaviour during meat-eating of both species. While Sonso chimpanzees have been observed to occasionally hunt blue duiker, they have never been observed to successfully hunt a red duiker in 27-years of almost continuous observation. Red duikers are large (around double the body mass of the blue duiker) and Waibira chimpanzees (including females who immigrated from Sonso) show similar behavioural levels of excitement during red duiker-meat eating, comparable to Sonso chimpanzees feeding on colobus. However, to date the number of hunts and meat-eating events recorded in Waibira remain relatively small; as a result it is very difficult to establish the absence of a particular behaviour which may be relatively infrequent, for example the abandoning of uneaten carcasses seen in Sonso.

Genetic differences between these two neighbouring communities are unlikely to explain the current variation in prey species hunted. There is evidence of regular genetic exchange between the groups, with at least four Sonso-born females known to have immigrated to the Waibira community in the past 5-years [69]. Ecological differences are harder to rule out. While the two communities share the same continuous forest habitat, it remains possible that minor variations in, for example: canopy structure [11, 18] may impact colobus hunting, or may impact encounter rates with the different prey species in the two communities. However, we also see a remarkably similarity in the pattern of hunting frequency and prey species hunted in Waibira today compared to that in an early study of Sonso hunting between 1994 and 2002 [5]. In both cases, hunting was reported only infrequently and with no bias towards colobus prey. While there has been limited illegal logging within the relatively protected range of the Sonso community, there is unlikely to have been a substantial change in canopy conditions within Sonso in the years before and after 2002. It seems more likely that human presence during the habituation of a new community contributes to the observed differences in colobus hunting: either between early and later Sonso data, or between current Waibira and Sonso data. The habituation hypothesis suggests that human presence may impact chimpanzee behaviour for an extended period of time, and should be examined as a potential ecological factor before cultural explanations can be considered.

Successful colobus hunts usually require multiple hunters [23, 43]. This requirement is less true for other prey species, which can be captured through opportunistic solo hunting. It is possible that the process of habituating chimpanzees disrupts complex social hunting attempts, and, in doing so, biases the prey-record in hunting observations towards solo-hunted species. It is difficult to test this hypothesis directly (as there are no prey-records from non-habituated communities); however, hunting, in particular group hunting, is frequently one of the last behaviours to be recorded in a new community. At all major chimpanzee study sites, hunting rates tended to be low during the first 5–10 years of observations (Tai [18]; Gombe [2]; Mahale: [8, 70]). At the same time, prey spectra were distributed across many species, particularly those that could be opportunistically grabbed by individual hunters [1, 8, 9]. At Sonso, where habituation started in 1990, only 17 hunts were recorded in the 8-years between 1994 and 2002 [5]. During this time the frequency of colobus to non-colobus hunting was the same as currently seen in Waibira today: around one third of all hunting attempts. It is only after 2002 that the Sonso prey bias towards colobus emerged. In Mahale researchers have described a very similar long-term shift in the prey profile, with an increase in the proportion of colobus prey only after 16-years of researcher presence (from 14% in the first 16-years to 56% in the next 7-years, and finally up to 83–84% consistently for the past 14-years [70]). In this study we compare hunting rates in the two halves of our 17-year data set–showing a striking difference in both the frequency of hunting and the distribution of prey species hunted. As a result, we caution against describing ‘typical’ hunting behaviour from the Budongo communities by averaging over long-term data, even once chimpanzees appear habituated. Hunting rates in Sonso in 1994–2002 were extremely low (average successful hunts 2 per year [5], estimated average total 5 per year). Over the past 14-years they are comparable to other east African communities (Sonso: average successful hunts = 12 per year, estimated average total = 27 per year); higher than some (e.g. Kanyawara [52]), lower than others (e.g. Kasekela [52]). In addition, even once habituation is extremely high, we see fluctuations in both the annual rate and apparent seasonal variation. Other factors, such as the presence of particular individuals who act as ‘impact hunters’ [52] or specific environmental events that may impact diet (e.g. El Nino years [71]), may also impact hunting behaviour and account for the variations observed in Budongo.

If the habituation hypothesis for the variation in colobus specialization seen in current Waibira and Sonso hunting (or early and late Sonso hunting) is correct, one striking question is, why does it take so long for groups to resume colobus hunting after the beginning of habituation? During the period of the first Sonso study (1994–2002) the majority of the mature individuals in the group were considered habituated, in that they could be followed without any obvious signs of stress or disruption to their daily activity [6]. Nevertheless, researchers with an interest in hunting behaviour who were present at this time rarely observed any signs of it (n = 17 in 8 years, [5]). Sonso chimpanzees predominantly hunt colobus prey as a group, whereas most hunts of other primates and all hunts of non-primate species were solo hunts. It would be interesting to examine whether early records of colobus hunting in Sonso contained a greater proportion of solo and opportunistic hunts compared to present day hunting behaviour. In Waibira, four of the six non-successful colobus hunts were all solo chases. However, it is difficult to obtain reliable data from early records because hunting behaviour, as opposed to subsequent meat-eating behaviour, is harder to observe during the early phases of habituation. One possible explanation is that the easier ‘fall-back’ option of solo non-colobus hunting becomes normalised for the generation of individuals who matured during the first few years of habituation. Some colobus hunting remains throughout but, given chimpanzees’ dietary conservatism [70, 72], it may take time for the prey bias to re-establish following the forced disruption from habituation and reincorporate species into their ‘prey profile’.

A concrete test of this would be to perform dietary analyses on faecal samples of non-habituated chimpanzee groups; if habituation does disrupt hunting we would predict that the prey preferences and hunting frequency in unhabituated groups should resemble those of communities in which hunting has resumed. Similarly, while not yet widely employed, it may be possible in the future to investigate unhabituated chimpanzee behaviour through the use of passive acoustic monitoring systems. Recently used to establish chimpanzee and other primate species density, ranging, and territory use [73, 74], the ability to triangulate the co-occurrence of both chimpanzee hunting barks and prey-species alarm calling may provide more detail on encounter rates and unsuccessful hunting attempts in unhabituated communities.

Neither ecological conditions nor the habituation hypothesis is likely to fully explain the absence of red duiker hunting in the Sonso communication. The Waibira group has repeatedly and successfully hunted red duiker, while the Sonso community has never been observed to do so. We found no differences in ungulate abundance between the sites, and although we were unable to distinguish Red duiker dung from Bushbuck dung reliably, Red duiker were encountered during surveying in both areas at a similar rate. As found in our own data, a recent large-scale survey suggested that there is little variation in duiker populations between the Sonso and Waibira areas [75].

Instead, this variation in duiker preference between communities may be a possible example of a ‘cultural phenomenon’ comparable with the group differences in food preferences already found between commonly available plant foods [76]. Interestingly, the four confirmed Sonso-born females who had recently emigrated to Waibira have all been observed begging for and feeding on red duiker meat, despite having never had the opportunity to do so in their natal community. While this is particularly surprising given the evidence for behavioural conservatism in chimpanzees [72, 77], recent research suggests that young immigrant females may rapidly conform to behavioural variations within their new community [78].

### Harassment and meat sharing

The apparent variation between neighbouring communities in meat-possession and sharing behaviour is of interest, but must also be interpreted with caution. A frequently encountered issue with long-term data is the tendency to ignore observations considered ‘typical’ and the non-systematic ad libitum nature of the entries. To date, our long-term data suggest that the ‘typical’ behaviour of the Sonso chimpanzee community when feeding on meat is that higher-ranking individuals harass and aggressively attack lower-ranking individuals to obtain possession of a carcass. Peaceful monopolization of a carcass by a low-ranking individual would be considered highly atypical in Sonso, but we find no recorded cases of it. We were unable to document the same pattern in the Waibira community, where lower-ranking sub-adult individuals maintained possession of any prey type for long periods with only non-contact or low-contact begging (classed as minimal harassment).

Here, as with prey-preference, it remains possible that differences in habituation could account for the observed behavioural differences. For example, sub-adult individuals tend to habituate more quickly than other age-classes [79] and are typically lower-ranking than adult individuals; however, higher-ranking individuals were present throughout our observations and in close proximity to the individual in possession of the carcass. It seems unlikely that a lower habituation level alone would inhibit aggressive behaviour towards lower-ranking individuals, but not inhibit the tendency to approach or remain in proximity to them. In addition, there are no records of similarly peaceful, extended, monopolization of large portions of a desirable carcass by low-ranking individuals in the presence of high-ranking individuals in the Sonso community, despite 182 hunting observations in an 18-year period. Even in the early period covered by the 2002 study, when habituation levels were lower, it was typical for high-ranking Sonso individuals to monopolise the carcass (Newton-Fisher, pers. comm.). A study of Fongoli chimpanzees in Senegal recently described observations of regular female meat monopolization, procured without actively hunting for it, and subsequent successful refusal of male begging behaviour [16]. The authors suggest that this supports a social bonding hypothesis, with the low levels of male aggression towards female meat possessors representing a long-term social investment [16]. If, as Pruetz et al. [16] suggest, this behaviour is part of a long-term social strategy, then there may be particular social and/or demographic factors that promote this, for example the ratio of adult males to adult females, resulting in its more regular use.

Our observations of the Sonso and Waibira community hunting behaviour suggest that 1) neither ecological nor genetic factors satisfactorily explain variation in chimpanzee hunting preferences, indicating that behavioural differences may represent responses to human observers or, in the case of red duiker prey, socially learned traditions and that 2) the energetic costs imposed by harassment alone may not provide a complete explanation of meat sharing in Budongo chimpanzees, to which social factors appear to make an important contribution.

## Supporting information

S1 Table Primate prey-species survey. Primate densities calculated from DISTANCE v7; 10km of transects surveyed per site, 20km total distance walked per year (2016 and 2017). *Note: given small sample size and high CV densities should be interpreted with caution (c.f. Plumtre, 2000).
(DOCX)
