# Supplementary material for: Variation in hunting behaviour in neighbouring chimpanzee communities in the Budongo forest, Uganda
Source: PLoS One. 2017 Jun 21;12(6):e0178065. doi: 10.1371/journal.pone.0178065 (PMC5479531; doi:10.1371/journal.pone.0178065)
Supplement: S1 Table — Primate densities calculated from DISTANCE v7; 10km of transects surveyed per site, 20km total distance walked per year (2016 and 2017). *Note: given small sample size and high CV densities should be interpreted with caution (c.f. Plumtre, 2000). (DOCX) [file pone.0178065.s001.docx]

**Table S1.** Primate prey-species survey. Primate densities calculated from DISTANCE v7; 10km of transects surveyed per site, 20km total distance walked per year (2016 and 2017). *Note: given small sample size and high CV densities should be interpreted with caution (c.f. Plumtre, 2000).

| **Species** | **Site** | **Density (ind km^-2^)** | **CV (%)** | **Density (groups km^-2^)** | **CV (%)** |
| --- | --- | --- | --- | --- | --- |
| Guereza colobus * monkey  (*Colobus guereza occidentalis*) | Sonso | 56 | 37.1 | 15.0 | 35.2 |
|  | Waibira | 40 | 35.9 | 11.25 | 31.9 |
| Blue monkey *  (*Cercopithecus mitis stuhlmanni*) | Sonso | 156.5 | 29.9 | 54.4 | 27.3 |
|  | Waibira | 42.2 | 39.6 | 17.7 | 35.1 |
| Red-tailed monkey *  (*Cercopithecus ascanius schmidti*) | Sonso | 55.6 | 34.7 | 36.8 | 32.2 |
|  | Waibira | 55.0 | 24.5 | 32.9 | 17.4 |
